# Isolation of viable *Babesia bovis* merozoites to study parasite invasion

**DOI:** 10.1038/s41598-021-96365-w

**Published:** 2021-08-20

**Authors:** Hassan Hakimi, Masahito Asada, Takahiro Ishizaki, Shinichiro Kawazu

**Affiliations:** 1 National Research Center for Protozoan Diseases, Obihiro University of Agriculture and Veterinary Medicine, Obihiro, Hokkaido 080-8555 Japan; 2 Department of Protozoology, Institute of Tropical Medicine (NEKKEN), Nagasaki University, Nagasaki, 852-8523 Japan; 3 Present Address: Department of Veterinary Pathobiology, College of Veterinary Medicine, Texas A&M University, College Station, TX USA

**Keywords:** Parasitology, Parasite biology

## Abstract

*Babesia* parasite invades exclusively red blood cell (RBC) in mammalian host and induces alterations to host cell for survival. Despite the importance of *Babesia* in livestock industry and emerging cases in humans, their basic biology is hampered by lack of suitable biological tools. In this study, we aimed to develop a synchronization method for *Babesia bovis* which causes the most pathogenic form of bovine babesiosis. Initially, we used compound 2 (C2), a specific inhibitor of cyclic GMP-dependent protein kinase (PKG), and a derivative of C2, ML10. While both inhibitors were able to prevent *B. bovis* egress from RBC and increased percentage of binary forms, removal of inhibitors from culture did not result in a synchronized egress of parasites. Because using PKG inhibitors alone was not efficient to induce a synchronized culture, we isolated viable and invasive *B. bovis* merozoites and showed dynamics of merozoite invasion and development in RBCs. Using isolated merozoites we showed that BbVEAP, VESA1-export associated protein, is essential for parasite development in the RBC while has no significant role in invasion. Given the importance of invasion for the establishment of infection, this study paves the way for finding novel antigens to be used in control strategies against bovine babesiosis.

## Introduction

*Babesia bovis* is a tick-borne intracellular protozoan parasite that causes the most pathogenic form of bovine babesiosis. *B. bovis* has a complex lifecycle with sexual and asexual replication in the tick vector and asexual multiplication in cattle as the intermediate host^1,2^. Asexual replication inside red blood cell (RBC) is responsible for parasite pathogenesis. The infection starts with the invasion of parasite sporozoites released from tick salivary glands followed by parasite growth, DNA replication, parasite multiplication, and finally egress from RBC. The egressed merozoites invade new RBCs and this cycle continues. These changes are likely driven by stage-specific gene expression in the parasite which needs experimental verification^3^. Currently, several *Babesia* species including *B. bovis* could be cultured in vitro^4^. Following the invasion, young merozoite is seen as ring (ring or trophozoite stage) and becomes paired or binary form following DNA replication which is considered as mature stage of the parasite. Parasite multiplication happens through binary fission which results in the production of two daughter cells^5^. Tetrad or maltese form which results from two rounds of DNA replication is not frequently seen in *B. bovis* and accounts for less than one percent of parasites in in vitro culture. Therefore, unlike *Babesia divergens* which makes a complex population structure^6^, the majority of *B. bovis* parasites are seen as ring or binary forms.

The parasite multiplies asynchronously in vivo and in the culture with the appearance of single, binary and free merozoites simultaneously^2^. In order to study parasite developmental stages such as egress or invasion which happens within minutes, it is needed to artificially induce synchrony in the culture. *Babesia* parasites increase RBCs permeability and change their density that could be used for enrichment and purification of infected RBCs (iRBCs) using percol-sorbitol or Histodenz density gradient centrifugation^7,8^. However, given that the parasite’s erythrocytic cycle is quite short (3.5–5 cycles per day for *B. bovis*)^9^, it is impossible to separate ring from binary forms using these techniques. To produce synchronously invaded parasite culture, free merozoites were used that were isolated from culture supernatant^10,11^, released by electroporation^9^, or cold treatment^12^. However, all these methods have drawbacks such as the need for large scale culture volume due to their low efficiency or parasite damage in case of using electroporation.

Inhibition of egress using chemicals targeting cyclic GMP-dependent protein kinase (PKG) was used to synchronize *Plasmodium falciparum* and *P. knowlesi*, the causative agents of human malaria^13–15^. In this study, initially we used compound 2 (C2) and a derivative of C2, ML10, the specific inhibitors of PKG, and showed that they can prevent egress and increase the proportion of binary forms. However, the removal of these compounds did not result in a synchronous egress of parasites. Therefore, we used filtration to mechanically release merozoites from erythrocytes and used these free merozoites to study the kinetics of RBC invasion by *B. bovis*. Using purified merozoites and conditional knockdown, we showed that BbVEAP is essential for *B. bovis* development in the RBC while has no significant role in invasion.

## Results

### Time-lapse imaging of *B. bovis*

**Figure 1:**
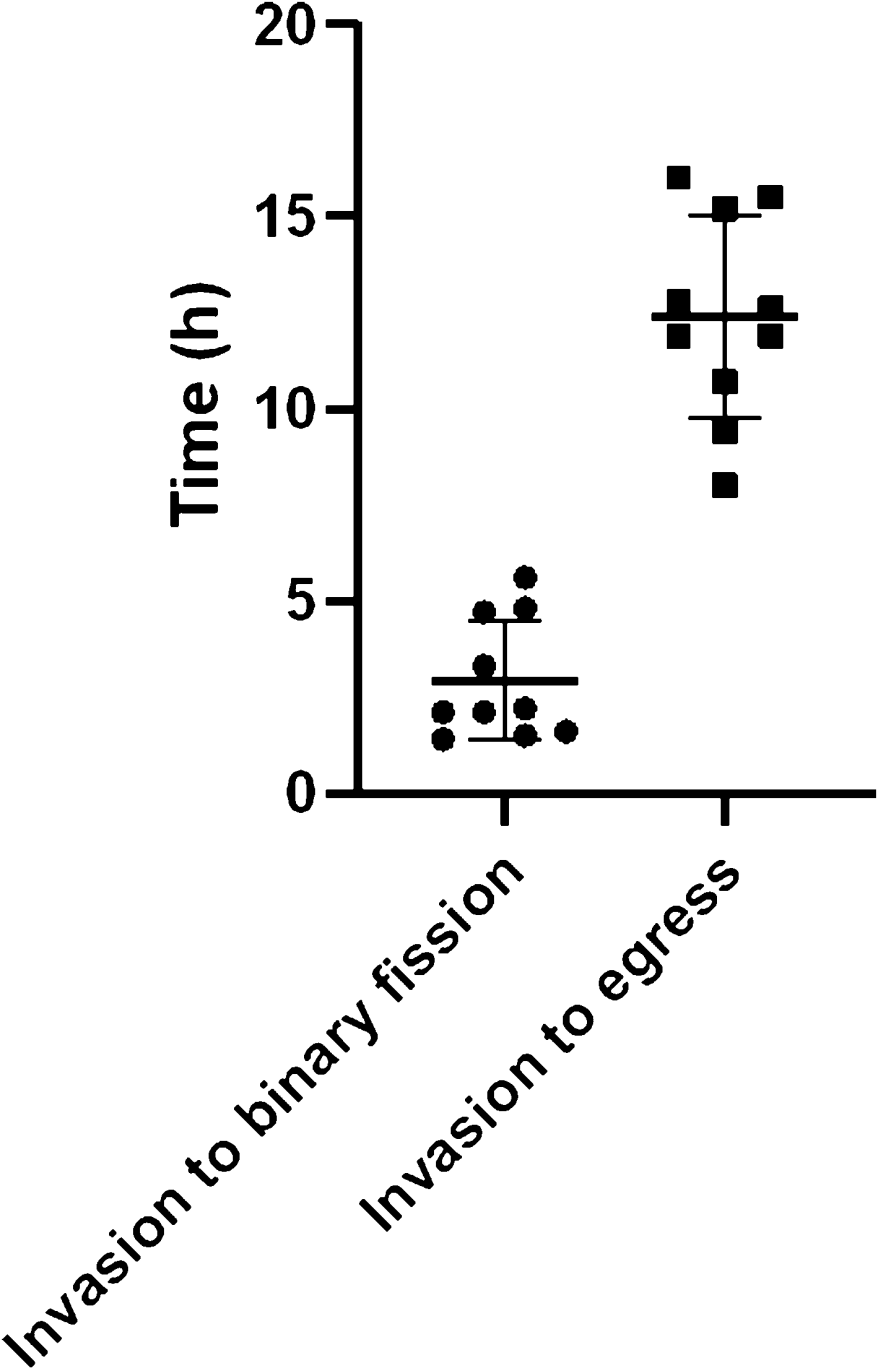
Time-lapse imaging of *B. bovis*. GFP-expressing *B. bovis* merozoites were observed over 24 h period and events following parasite egress and subsequent invasion were witnessed (n = 10).

To visualize the events during *B. bovis* development inside RBC and estimate one erythrocytic cycle, we performed time-lapse imaging using GFP-expressing parasites. This parasite line was produced by replacing *tpx-1* open reading frame (ORF) with *gfp* and was shown to have no growth defect in the culture^16^. We used confocal microscopy by taking images of growing parasites with 30 s interval over 24 h (video 1). We followed up the parasites from invasion into and egress from RBCs. As shown in Fig. 1, the average of one complete cycle was 12.4 h (n = 10; S.D. ± 2.6 h). We were able to observe transition state from ring to binary form at 2.9 h post invasion (n = 10; S.D. ± 1.6 h). Additionally, some ring forms did not develop to binary forms during imaging time which may indicate the existence of gametocytes, dormant *B. bovis*, or the adverse effect of hypoxemia on parasite viability and development that needs future validation (Video 1, bottom left).

### Egress arrest using PKG inhibitors

**Figure 2:**
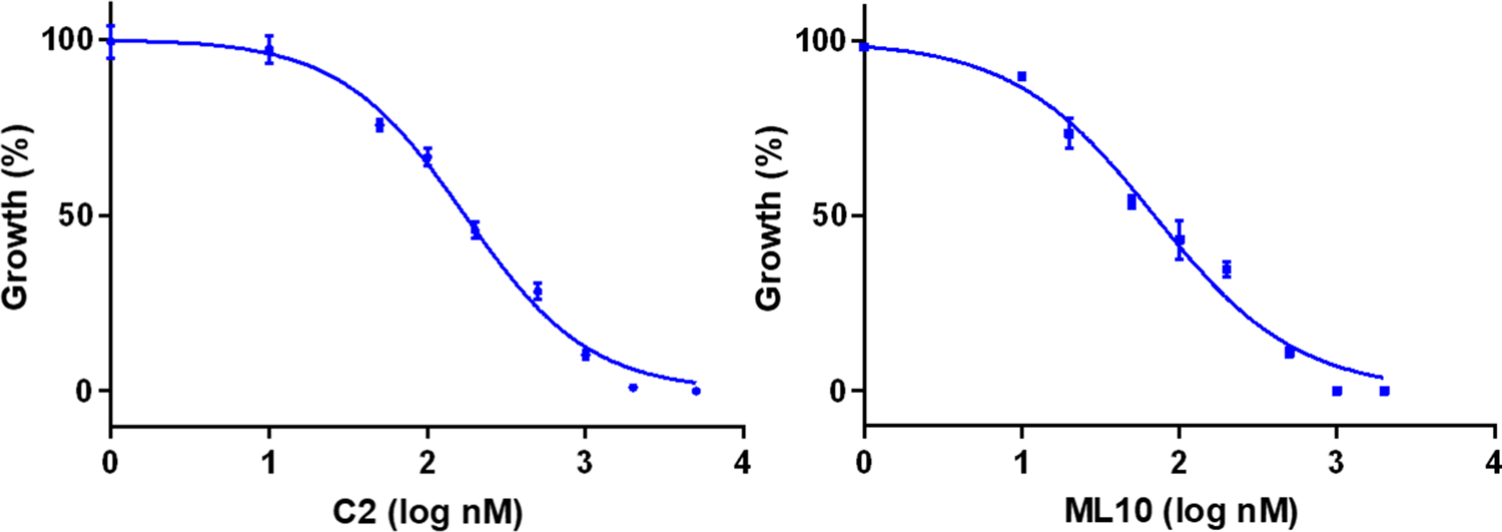
Dose–response curve of C2 and ML10 for *B. bovis*. The parasites were cultured in presence of different concentrations of C2 or ML10. Data are shown as mean ± SEM of triplicate culture.

**Figure 3:**
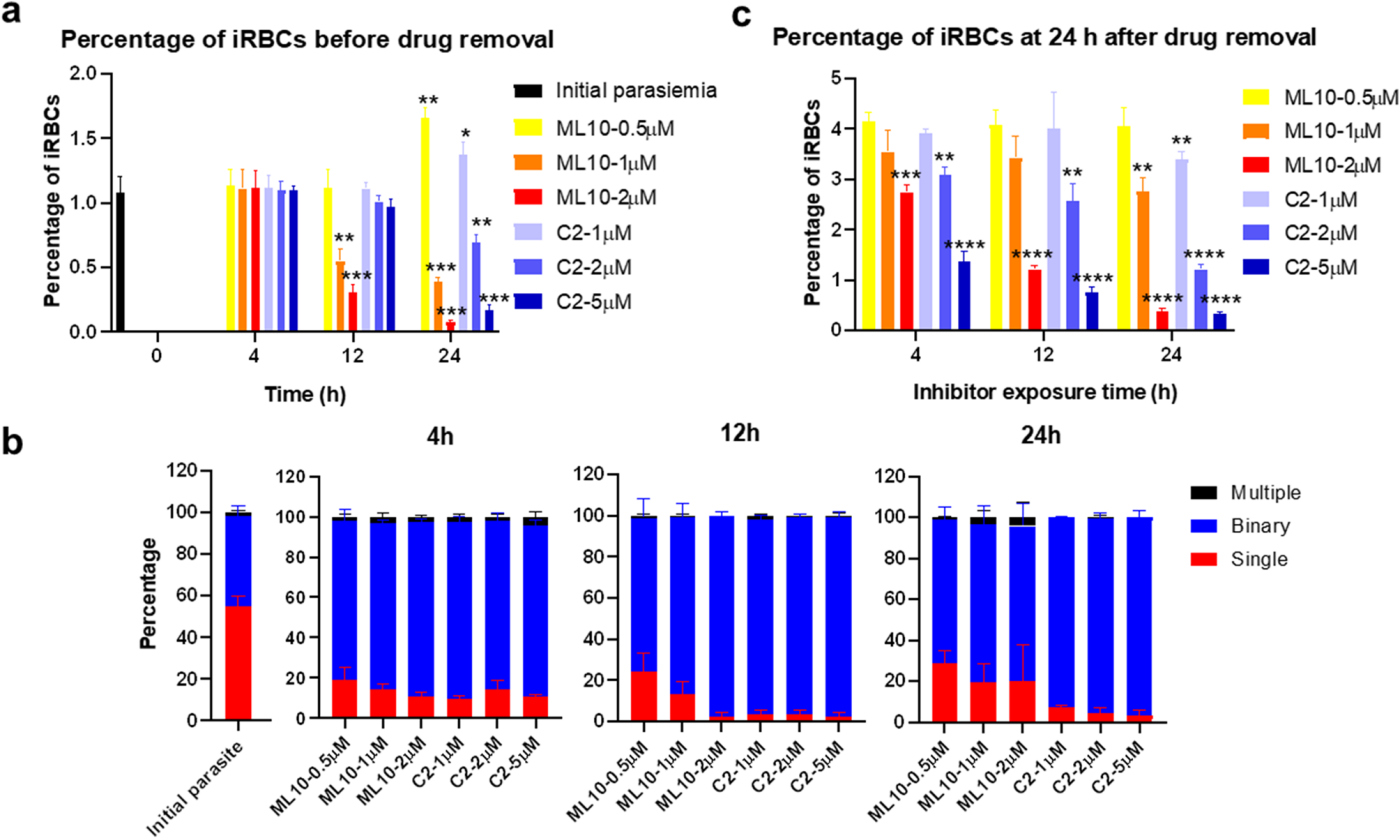
Impact of ML10 and C2 concentration and length of exposure on egress block and parasite viability. (**a**) Parasites were cultured in presence of 0.5, 1 or 2 μM of ML10 or 1, 2 or 5 μM of C2 for 4, 12, or 24 h. The initial percentage of iRBCs was ~ 1% and percentages of iRBCs in presence of drugs were calculated. The data are shown as mean ± S.D. of triplicate culture. Statistical comparisons were done between each group and initial percentage of iRBCs. (*P < 0.05; **P < 0.01; ***P < 0.001; determined by unpaired *t* test). (**b**) The proportion of ring, binary, and multiple stages in presence of ML10 or C2 for 4, 12, or 24 h. The data are shown as mean ± S.D. of triplicate culture. (**c**) Cultures that were exposed to different concentrations of ML10 or C2 for 4, 12, or 24 h were washed and allowed to grow in fresh medium for 24 h. The statistical significance of the difference between each group and parasite treated for 4 h of 0.5 μM of ML10 or 1 μM of C2 determined by unpaired *t* test. (**P < 0.01; ***P < 0.001; ****P < 0.0001). The data are shown as mean ± S.D. of triplicate culture.

To validate the application of PKG inhibitors, C2 and ML10, for synchronization of *B. bovis*, we first determined EC_50_ of these compounds. The wild type parasites were cultured in the presence of C2 or ML10 in a 72 h growth inhibition assay. The EC_50_ of C2 and ML10 was 172 ± 29.4 nM and 69.9 ± 18.7 nM, respectively (Fig. 2). To investigate the proper concentration of drugs and the length of exposure time, we incubated the parasites with different concentrations of ML10 or C2 for 4, 12 or 24 h (Fig. 3a). 0.5 µM of ML10 for 4 and 12 h arrested the egress of parasites and increased the proportion of binary forms (Fig. 3a,b). Exposure to 1 or 2 µM of ML10 for 4 h also prevented egress and increased the percentage of binary forms. However, longer exposure to 12 or 24 h with 1 or 2 µM of ML10 affected viability of parasites and significantly decreased percentage of iRBCs (Fig. 3a). In regards to C2, 1, 2 or 5 µM of the drug for 4 or 12 h was able to prevent egress and increased the proportion of binary parasites up to 95% which was higher than ML10 (Fig. 3a,b). Exposure to 2 and 5 µM of C2 for 24 h decreased the viability of parasites seen as a significant decline in percentage of iRBCs (Fig. 3a). While both drugs were able to arrest egress and increase binary forms, we decided to validate whether this arrest is reversible and how long the arrested parasites remain viable. Following exposure to different concentrations of drug for 4, 12 or 24 h, the iRBCs were pelleted and incubated in fresh medium and percentage of iRBCs was calculated at 24 h post drug removal. Incubation with 0.5 µM ML10 up to 24 h or 1 µM C2 for 4 h and 12 h had a negligible effect on the parasite viability (Fig. 3c). Similarly, exposure to 1 µM of ML10 up to 12 h had a minor effect on parasite growth while longer incubation time or increasing drug concentrations significantly declined parasite viability (Fig. 3c). We decided to further validate whether 0.5 µM of ML10 or 1 µM of C2 could be used for *B. bovis* synchronization.

**Figure 4:**
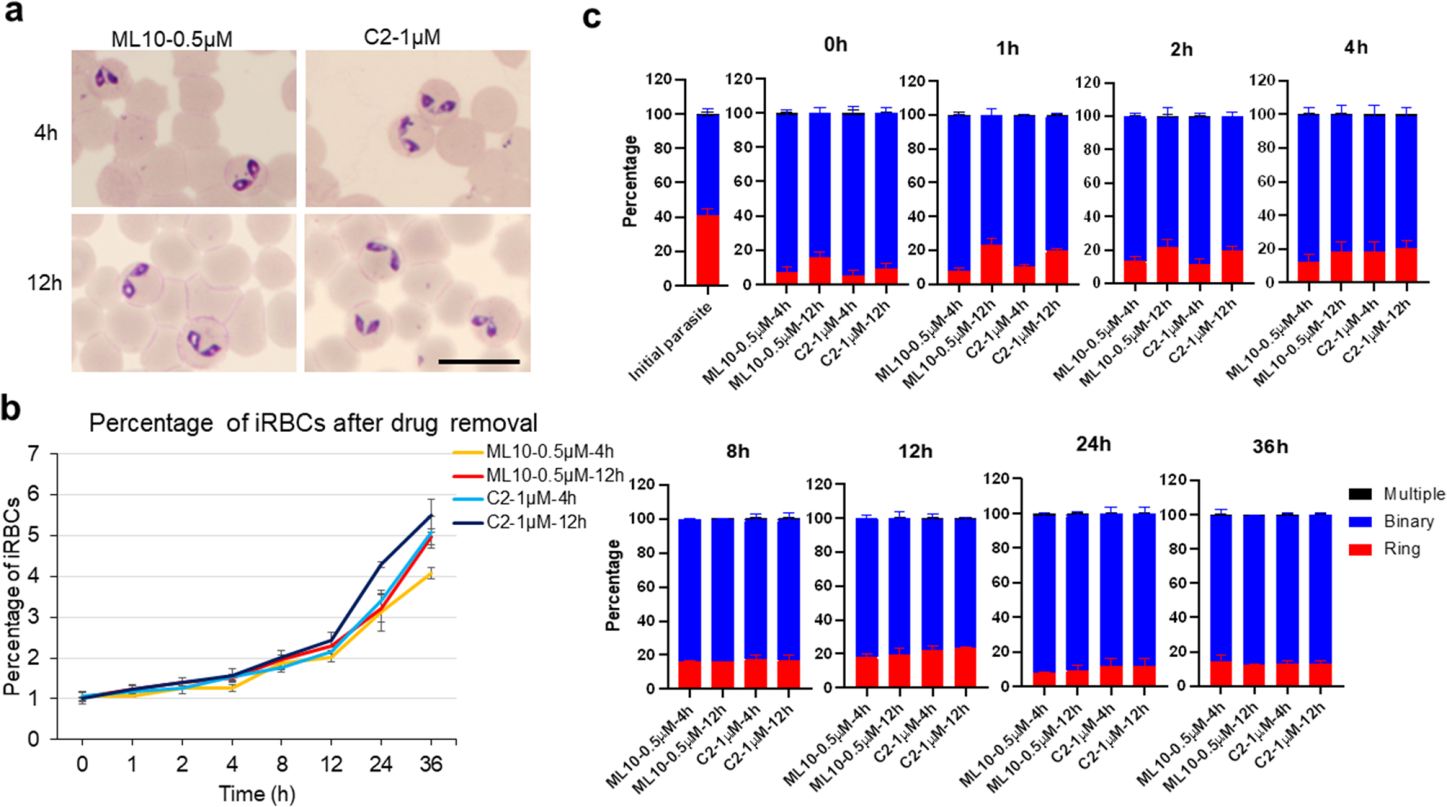
*Babesia bovis* growth and egress following removal of C2 and ML10. (**a**) The Giemsa-stained smears were prepared before drug removal to validate the effects of drugs on parasite morphology. Scale bar = 10 µm. (**b**) Parasites that had been arrested in the culture in the presence of 0.5 μM of ML10 or 1 μM of C2 for 4 or 12 h were washed and transferred to fresh medium to allow egress and invasion of new RBC for 36 h. The data are shown as mean ± S.D. of triplicate culture. (**c**) Proportion of ring, binary, and multiple stages in initial parasites, at the time and following removal of ML10 or C2 were calculated for 36 h (mean ± S.D. of triplicate culture).

The parasites were incubated in the presence of ML10 or C2 for 4 or 12 h. 0.5 µM of ML10 or 1 µM of C2 did not cause a noticeable change in the parasite’s morphology (Fig. 4a). The drugs were removed and the cultures were monitored for a further 36 h. Following drug removal, the percentage of iRBCs increased gradually indicating that the egress arrest was reversible (Fig. 4b). However, we cannot exclude the possible adverse effects of these drugs on parasites since we were not able to include a proper control in the absence of a synchronization method for *B. bovis*. While incubation with C2 or ML10 increased the proportion of binary forms, drug removal resulted in a gradual and not synchronized egress of parasites (Fig. 4c). These results indicate while PKG is important for egress of *B. bovis*, other factors such as calcium-dependent protein kinase (CDPK) and cAMP-dependent kinase (PKA) may contribute^3,17^. Thus, PKG inhibitors alone are not sufficient to synchronize *B. bovis* in the culture.

### Isolation of viable and invasive *B. bovis* merozoites

**Figure 5:**
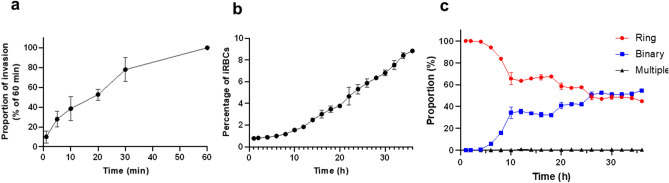
Invasion kinetics of *B. bovis* filter isolated merozoites. (**a**) The proportion of merozoites that successfully invaded erythrocytes is plotted over time relative to a 60-min maximum incubation (mean ± S.D. of three independent experiments in triplicate culture). (**b**) Parasite growth over 36 h time course. Smears were prepared every 2 h and data are shown as mean ± S.D. of triplicate culture. (**c**) Proportion of ring, binary, and multiple stages following invasion (mean ± S.D. of the triplicate experiment).

Because PKG inhibitors alone were not effective to prepare a synchronized culture, we decided to mechanically release the merozoites with filtration to synchronize parasites at free merozoite stage and study the parasite invasion. Considering 1.5–1.9 μm size of *B. bovis* merozoite^18^, we decided to use 2 μm filter for merozoite purification. Given that following one-time filtration some intact RBCs were seen in Giemsa-stained smear, we performed double filtration which reduced the number of intact noninfected and iRBCs (Sup. Fig. 1). Next, we investigated the parasite invasion capacity following filtration. Initially, we explored invasion of the purified merozoites following incubation with noninfected bovine RBCs over 1 h time course (Fig. 5a). The invasion of *B. bovis* merozoites increased gradually over time and most invasion events happened within 30 min (~ 80% of total invasion) while merozoites kept their invasion capacity up to 1 h.

The growth of *B. bovis* merozoites following invasion was monitored by Giemsa staining for a course of 36 h (Fig. 5b,c). In the in vitro culture, the majority of parasites exist as ring or binary forms. Following merozoite invasion, all parasites were ring form up to 4 h and gradually binary form parasites started to appear from 6 h post invasion. The proportion of binary forms gradually increased and became dominant in the culture at 26 h when the percentage of iRBCs reached more than 5% (Fig. 5b,c). These results suggested that purified merozoites were viable and grow normally in the culture and could be used to study RBC invasion.

### Induced knockdown of BbVEAP did not affect RBC invasion but arrested parasite development

**Figure 6:**
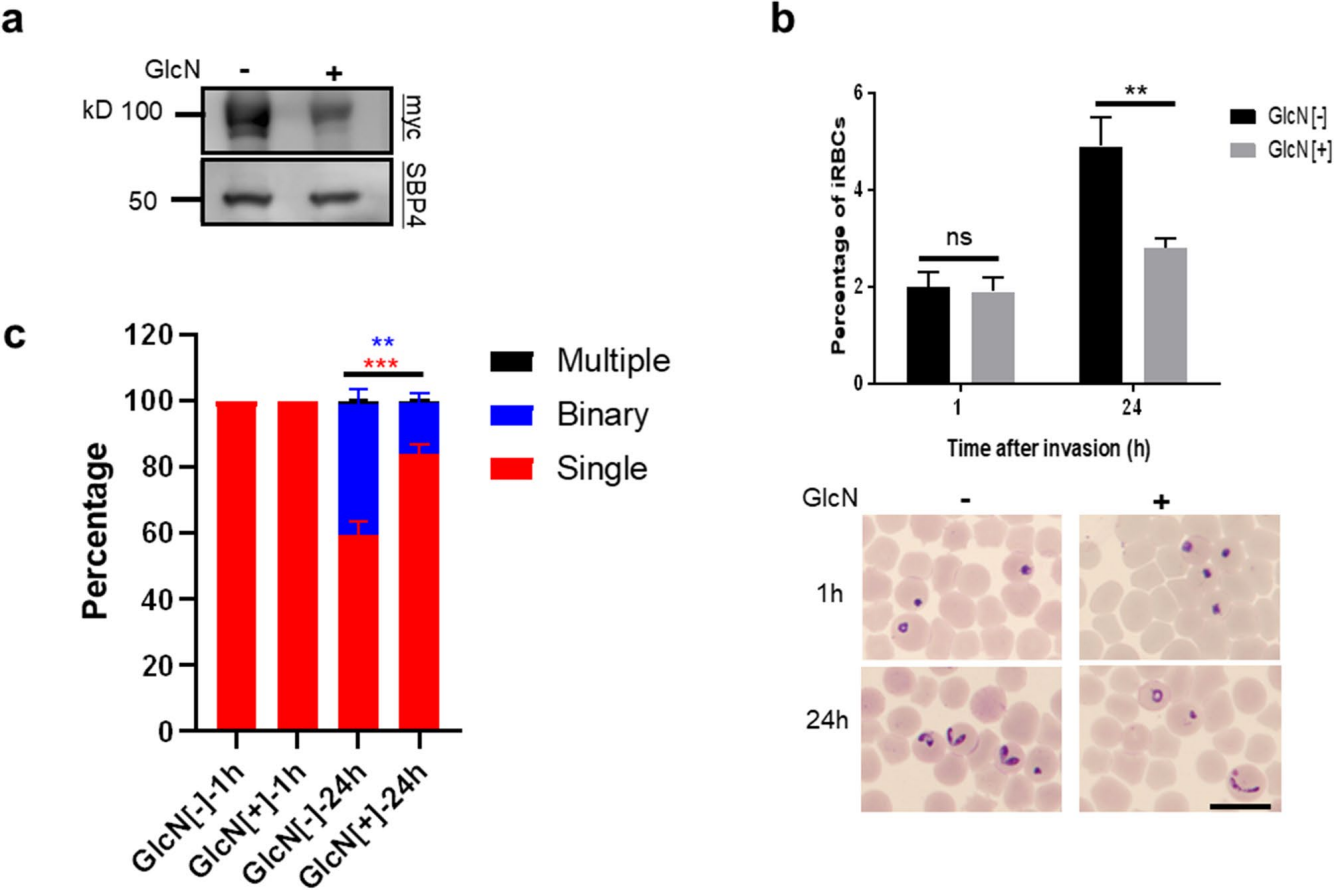
BbVEAP knockdown did not affect parasite invasion. (**a**) Western blot analysis of myc-*glmS* expressing *B. bovis* in the presence or absence of glucosamine (GlcN). Anti-SBP4 antibody was used to detect SBP4 protein as a loading control. The image is representative of three independent experiments. Full-length blots are presented in Sup. Fig. 3. (**b**) The myc-*glmS* expressing merozoites in the presence or absence of GlcN for 24 h were filter isolated and an invasion assay was performed. Percentage of iRBCs was measured at 1 h and 24 h after the invasion. The data are shown as mean ± S.D. of three independent experiments in triplicate culture. (ns, not significant; **P < 0.01 determined by unpaired *t* test). Scale bar = 10 µm. (**c**) Proportion of ring, binary, and multiple stages in parasites in the absence or presence of GlcN at 1 h or 24 h following invasion (mean ± S.D. of three independent experiments in triplicate culture. **P < 0.01; ***P < 0.001 determined by unpaired *t* test).

Recently, we found a novel spherical body protein, BbVEAP, that is exported to RBC during parasite development^8^. Because the export of VESA1, Variant Erythrocyte Surface Antigen 1, as a ligand for cytoadhesion was dependent on the expression of BbVEAP, this protein was named as VESA1-export associated protein, BbVEAP^8^. Additionally, conditional knockdown of BbVEAP reduced *B. bovis* growth suggesting an important role of this protein for parasite development^8^. To investigate whether this growth defect is due to decreased invasion and/or parasite development in the RBC, we used *bbveap glmS*-myc tagged parasites^8^. In this parasite, myc-*glmS* sequence was inserted at the 3’ end of *bbveap* ORF^8^*.* The self-cleaving *glmS* ribozyme could be activated by glucosamine-6-phosphate (GlcN) and results in degradation of mRNA and knock down of target protein^19^. It was shown that 5 mM GlcN over 3-day time course had no significant effects on *B. bovis* growth^8^*.* Thus, *bbveap glmS*-myc tagged parasites were treated with 5 mM of GlcN for 24 h, merozoites were purified, invasion assay was performed, and parasites growth was monitored for 24 h post invasion (Fig. 6). GlcN treatment resulted in roughly 66% reduction of BbVEAP expression which was confirmed by Western blot analysis (Fig. 6a). While VEAP knockdown parasites kept their invasion ability similar to control parasites, they showed a significantly lower percentage of iRBCs at 24 h post invasion with a higher proportion of ring forms, and a decreased percentage of binary forms (Fig. 6b,c). Indirect immunofluorescence antibody test at 24 h post invasion confirmed specific knockdown of BbVEAP while the expression of control protein, spherical body protein 4 (SBP4) was unchanged (Sup. Fig. 2). Altogether, these results suggest that the lower growth rate of BbVEAP knockdown parasites is due to developmental defect and not impaired invasion ability.

## Discussion

Dissecting the developmental stages of *B. bovis* inside RBC is hampered by the lack of high throughput synchronization methods. The chemical arrest of egress using PKG inhibitors was successfully used to synchronize *P. falciparum* and *P. knowlesi*^15^. PKG in malaria parasites is responsible for the initiation of a signaling cascade that results in the lysis of parasitophorous vacuole membrane (PVM) and subsequently RBC membrane and parasite egress^14^. C2 and ML10 were used to reversibly block PKG and prevent malaria parasite egress^20^. Removal of these inhibitors results in merozoite release within minutes^15^. *B. bovis* has a single PKG with a conserved gatekeeper T618 (BBOV_I004690). In our study, C2 and ML10 were able to prevent parasite egress and increase the proportion of binary forms. While the arrested parasites were able to grow following inhibitor removal, the egress from RBCs was gradual and took several hours. This difference in egress of *B. bovis* with *Plasmodium* could be due to the lack of PVM and the difference in signaling cascade responsible for egress. *B. bovis* PVM is ruptured few minutes after invasion^21^ which may cause a difference in egress signaling cascade in comparison with malaria parasites that maintain PVM during development in the RBC. Additionally, in our study, long exposure of *B. bovis* to PKG inhibitors did not result in induction of multiple fission^5^ and production maltese form or iRBCs with more than two parasites. This is similar to *Plasmodium* that longer exposure of PKG inhibitors decline viability and did not increase merozoite numbers per schizont^15^. Treatment of *B. bovis* with a bumped kinase inhibitor, RM-1-152, resulted in egress arrest and an increase in the number of parasites per RBC^22^ which is similar to the egress block in *Toxoplasma gondii*^23^. However, the targeting kinase by RM-1-152 is unclear to delineate the egress block phenotype in *B. bovis* and needs further investigation^3^. Egress and growth arrest of *B. bovis* in the presence of PKG inhibitors confirmed PKG as a promising target for the treatment of babesiosis and these compounds could be used to study egress in these parasites.

Because application of PKG inhibitors did not produce a synchronized *B. bovis* culture, we decided to mechanically release the merozoites from RBC. Isolation of free merozoites has been used for culture synchronization and studying invasion of malaria parasites and *B. divergens*^6,24–26^. The purified *B. bovis* merozoites were able to invade even up to 1 h after mixing with bovine RBCs. However, 80% of total invasion happened within 30 min. This ability to keep the invasion capacity for a long duration is similar to *B. divergens* and *P. knowlesi*^6,26^ and different from *P. falciparum* merozoites which are viable for a few minutes^24^. To have a shorter window of invasion and a tighter synchronized culture, it is possible to prevent further invasion using invasion inhibitors such as heparin^27^. The average doubling time was roughly 10 h for the purified merozoites that invaded RBCs while this was 12.4 h in our time-lapse imaging experiment. This difference could be due to the difference in culture conditions and the number of parasites that were visualized. Purified *B. bovis* merozoites started growing upon invasion and lost their synchronicity within one cycle (Fig. 5c). This is partially due to the short lifecycle of *B. bovis* and their dynamic growth. However, the simplicity and high throughput efficiency of merozoite purification in this study and our established time-lapse imaging can make this method as a routine and valuable tool to study *B. bovis* invasion and its development in the RBC.

We used purified merozoites to study the function of BbVEAP, a recently identified secreted protein into the RBC cytoplasm^8^. Previously, we have shown that BbVEAP is involved in ridge formation, VESA1 export and expression on the surface of iRBC, and cytoadhesion of iRBCs to endothelial cells^8^. While induced knockdown of BbVEAP reduced percentage of iRBCs, whether this reduction is due to invasion defect is not known. Here we have shown that BbVEAP knockdown merozoites have no defect in invasion ability and reduction of percentage of iRBCs is due to impaired development in the RBC. This gene is conserved across piroplasmida and is upregulated in *B. bovis* blood stage^28^ which suggests a conserved important function for BbVEAP during development in the RBC. Identification and characterization of interacting proteins with BbVEAP may shed light on BbVEAP role during parasite development.

In conclusion, the methodology of merozoite purification introduced in this paper is robust and could be a valuable tool to study *B. bovis* development in the RBC.

## Methods

### Parasite culture

*B. bovis* Texas T2B strain^29^ was maintained in culture using a microaerophilic stationary-phase culture system composed of bovine RBCs at 10% hematocrit and GIT medium (Wako Pure Chemical Industries, Japan). For time-lapse imaging, a hybriwell chamber with 13 mm diameter and 0.15 mm depth (Grace BioLabs, USA) was used. Cells with 3% packed cell volume (PCV) from parasite culture were loaded, the chamber was sealed, and cells were viewed at 37 °C using a confocal laser-scanning microscope (A1R; Nikon, Japan). The images were taken using DIC and laser 488 nm for GFP at 30 s interval over 24 h.

### Measurement of EC_50_ of PKG inhibitors

C2 and ML10 were received from LifeArc, dissolved in DMSO at 2 mM concentration, and kept at − 30 °C. *B. bovis* wild type parasites were cultured in the absence or presence of different concentrations of C2 or ML10. The initial percentage of iRBCs was 0.05% and parasites were cultured in triplicate for 3 days with daily culture medium change. Percentage of iRBCs was calculated by examining at least 10,000 RBCs on thin smears prepared on day 3.

### Merozoite purification

IRBCs from cultures with 5% percentage of iRBCs or more were pelleted at 600×*g* for 5 min. IRBCs are resuspended in PBS at 10% PCV and were filtered twice manually at room temperature through a filter unit with a 2 µm pore size and 25 mm diameter (Isopore), spun down and washed once with PBS (1000 g, 5 min, RT). The filtration was done slowly using a 20 ml syringe at ~ 5 mL/min. The merozoites were quantified using a hemocytometer and used for invasion assay with a ratio of 1:10 to bovine RBCs. Merozoites and bovine RBCs at 10% hematocrit were mixed at 250 rpm for 10 min at 37 °C and transferred to a culture incubator.

### Western blotting

IRBCs were treated with 0.2% saponin and proteins were extracted using 1.0% Triton-X 100 (w/v) in PBS containing protease inhibitor cocktail (Complete Mini, Roche) at 4 °C for 1 h. The protein fractions were separated by electrophoresis using 5–20% SDS–polyacrylamide gradient gel (ATTO, Tokyo, Japan) in a reducing condition and transferred to polyvinylidene difluoride membranes (Clear Blot Membrane-P, ATTO, Tokyo, Japan). The membrane was probed with rabbit anti-myc polyclonal antibody (1:500; ab9106, Abcam, UK) or rabbit anti-SBP4 polyclonal antibody (1:1000)^30^ at 4 °C overnight. The membrane was incubated with HRP-conjugated goat anti-rabbit IgG (1:8000; Promega, USA) as the secondary probe. Protein bands were visualized using Immobilon Western Chemiluminescent HRP substrate (Merck Millipore) and detected by ImageQuant LAS 500 (GE healthcare).

### Indirect immunofluorescence antibody test

Thin blood smears from cultured parasites were prepared, air-dried and fixed in a 1:1 acetone:methanol mixture at − 30 °C for 5 min^31^. Smears were blocked with PBS containing 10% normal goat serum (Invitrogen) at 37 °C for 30 min and immunostained with mouse anti-myc monoclonal antibody (9B11, Cell Signaling Technology) at 1:500 dilution in PBS supplemented with 0.05% Tween-20 and incubated at 4 °C overnight. Double immunostaining of smears was done with rabbit anti-SBP4 at 1:1000 dilution. The smears were incubated with Alexa Fluor 488-conjugated goat anti-mouse or Alexa Fluor 594-conjugated goat anti-rabbit IgG antibody (1:500; Invitrogen) at 37 °C for 30 min. Nuclei were stained by incubation of smears with 1 μg/mL Hoechst 33342 solution. The smears were examined using a confocal laser-scanning microscope (CS-SP5, Leica Micro-system, Wetzlar, Germany).

### Statistical analysis

Statistical analysis was performed using GraphPad Prism 8. Percentage of iRBCs and proportion of parasite stages were plotted and evaluated using unpaired *t*-test. The values were considered significantly different if *P*-value was below 0.05.

## Supplementary Information

Supplementary Information 1.

Supplementary Video S1.
