# Supplementary material for: Isolation of viable Babesia bovis merozoites to study parasite invasion
Source: Sci Rep. 2021 Aug 20;11:16959. doi: 10.1038/s41598-021-96365-w (PMC8379152; doi:10.1038/s41598-021-96365-w)
Supplement: Supplementary file 1 — Supplementary Information 1. [file 41598_2021_96365_MOESM1_ESM.pdf]

Supplementary information

Video 1. Live imaging microscopy of GFP-expressing *B. bovis*. Parasites were imaged at a rate of 1 frame/30 seconds. The egress of a maltese stage parasite and invasion into new RBC, binary fission, and its egress could be seen.

Sup Fig 1. **Evaluation of filter isolated merozoites.** Free merozoites after one (a) or two rounds of filtration (b). Red arrows show intact iRBCs.

Sup Fig 2. Indirect immunofluorescence microscopy of BbVEAP knockdown parasites. Indirect immunofluorescence microscopy test of BbVEAP-myc-*glmS* parasite in the absence (-) or presence (+) of GlcN at 24 h after invasion (α-myc, green; α-SBP4, red). The parasite nuclei were stained with Hoechst 33342 (blue). Scale bar = 5 µm.

Sup Fig 3. Western blot analysis of myc-*glmS*-expressing *B. bovis*. Full-length blots of Fig 6a.

Sup Fig. 1

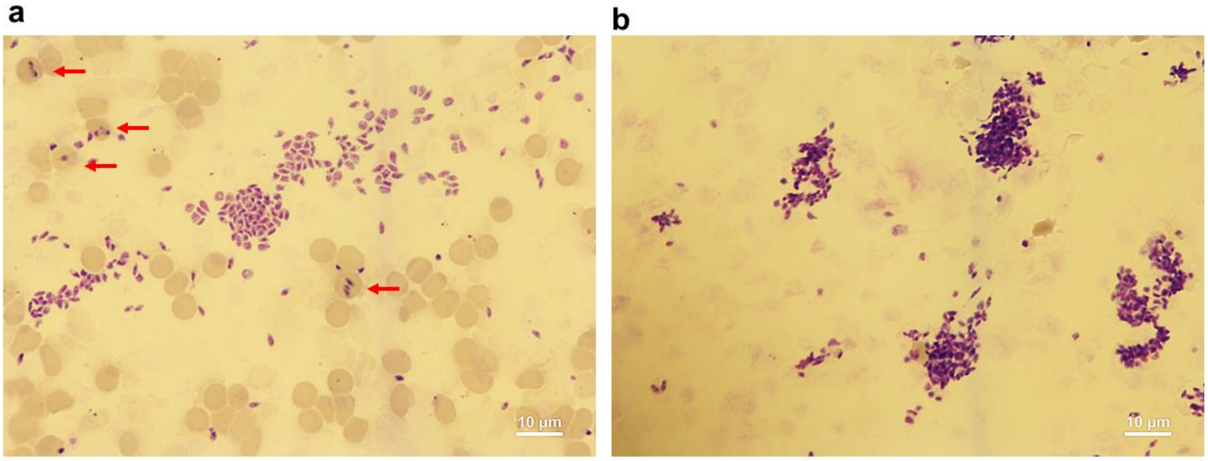

Sup Fig. 2

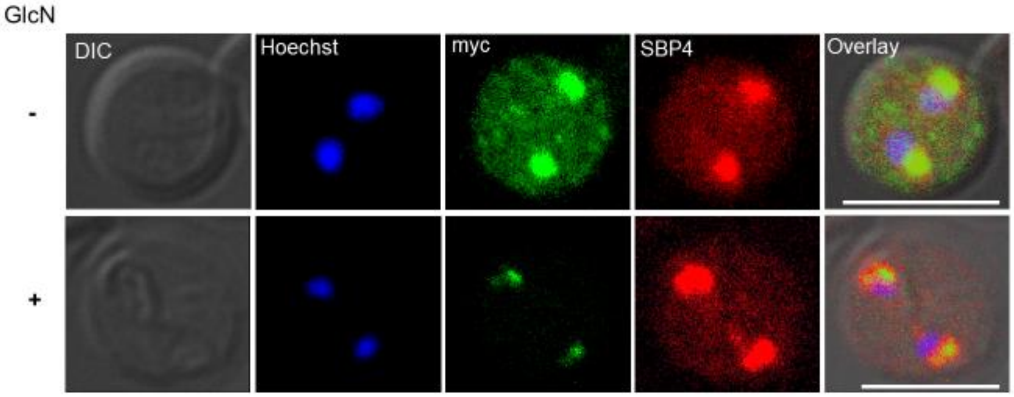

Sup Fig. 3

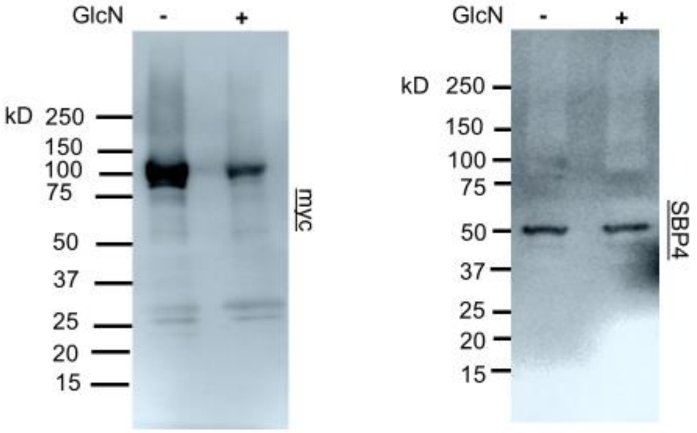

GlcN
-
+
kD
250
150
100
75
50
37
25
20
15
myc
GlcN
-
+
kD
250
150
100
75
50
37
25
20
15
SBP4
